# Recent Experiments with Laughing Gas

**Published:** 1879-07

**Authors:** 


### ARTICLE VII.

### *Recent Experiments with "Laughing Gas."*

Protoxide of nitrogen, or "laughing gas," the anæsthetic properties of which were discovered by Sir Humphry Davy, is used at the present time by a very large number of dentists for producing insensibility during the process of extracting teeth. But this insensibility cannot be prolonged for any great length of time owing to the fact that asphyxia is liable to supervene. For this reason, American surgeon dentists have succeeded in performing lengthy operations by means of this gas, only in producing short, but repeated anæsthesia, separated by intervals of sensibility. The reason of this is that anæsthesia can only be produced by making the patient respire pure protoxide of nitrogen, without any admixture of air; the result is that asphyxia is a concomitant of anæsthesia. The celebrated physiologist, M. Paul Bert, has recently been experimenting on this subject with a view of discovering some means of overcoming the latter difficulty, and obtaining from laughing gas anæsthetic effects that may be indefinitely prolonged, while at the same time they shall be absolutely free from any dangers arising from asphyxia. The results of his investigations were presented in a paper read before the French Academy of Sciences on the 11th of November. It is proper to remark here that M. Bert's experiments were made upon animals solely. The fact that protoxide of nitrogen must be administered in a pure state signifies that the tension of this gas, in order that it may penetrate in sufficient quantity into the organism, must be equal to one atmosphere. In order to obtain it, under the normal pressure, it is necessary that the gas be in the proportion of 100 per cent. But if we suppose the patient placed in an apparatus where the pressure may be carried up to two atmospheres, we shall be able to submit him to the desired tension in making him respire a mixture of 50 per cent protoxide of nitrogen and 50 per cent air; we ought then to obtain anæsthesia, while at the same time we maintain the normal quantity of oxygen in the blood, and consequently preserve the normal conditions of respiration. And this is just what happens. In M. Bert's experiments he tells us that he entered an apparatus constructed for the purpose, and there under an increase of pressure of one-fifth of an atmosphere he caused a dog to respire a mixture of five-sixths of protoxide of nitrogen and one-sixth oxygen —a mixture in which, as may be seen, the tension of the laughing gas is precisely equal to one atmosphere. Under such conditions the animal fell, in one or two minutes, into a complete state of anæsthesia, and had it not been for its respiration, which was executed with perfect regularity, it would have seemed to be dead. This state was found to last for an entire hour without the least change; the blood preserving its red color, the heart its regular beats, and the temperature its normal degree. During this whole period, all those phenomena of life called vegetative remained intact, while all those of animal life were absolutely annulled. When the bag containing the mixed gases was at length removed, the animal was observed, at the third or fourth inspiration of pure air, to suddenly recover its sensibility, will, intelligence, and natural friskiness. This rapid return to a normal state, so different from what is observed on the administration of chloroform, is due to the fact that laughing gas does not, like the latter, form chemical combinations in the organism, but is simply dissolved in the blood. As soon as none of it longer exists in the inspired air, it rapidly escapes from the system, through the lungs, as analysis of the blood have proved. As a result of many very careful experiments, M. Bert states that he feels himself authorized to maintain that the use of protoxide of hydrogen is perfectly harmless; and furthermore, he strongly recommends surgeons to use this gas under pressure, with a view of obtaining its anæsthetic effects as long as possible. By measuring, as above indicated, the barometric pressure and the centesimal composition of the mixture, so as to have for the protoxide of nitrogen the tension of the atmosphere, and for the oxygen at least the normal tension in the air, they will obtain a state of insensibility and a muscular resolution as complete as they desire, with an immediate return to sensibility and perfect state of well being, on removal of the anæsthetic agent. The sole difficulty in the way relates to the apparatus necessary to make the application of the anæsthetic under tension. For army purposes this is insuperable, but in cities the difficulty is easily remedied, for in such places compressed air baths are always obtainable, and in fact might be easily constructed in the surgical wards of hospitals at small expense. This, however, is a matter of secondary consequence, the solution of which remains with surgeons themselves; to whom, as well, it belongs to resolve the numerous questions of detail that always accompany the application of a new therapeutic agent.—*Scientific American.*

---

